# ERA5–Drought: Global drought indices based on ECMWF reanalysis

**DOI:** 10.1038/s41597-025-04896-y

**Published:** 2025-04-14

**Authors:** Jessica Keune, Francesca Di Giuseppe, Christopher Barnard, Eduardo Damasio da Costa, Fredrik Wetterhall

**Affiliations:** European Centre for Medium-Range Weather Forecasts, Shinfield Park, RG2 9AX Reading, UK

**Keywords:** Natural hazards, Hydrology

## Abstract

Droughts are increasingly intensified by human-induced climate change and pose a growing threat to society. Thus, enhancing our capabilities to monitor drought occurrence and intensity is crucial. This paper introduces a new dataset of drought indices derived from the 5th generation ECMWF reanalysis system (ERA5), which offers long-term monitoring of the global climate in both deterministic and probabilistic forms. This global dataset is freely accessible through an ECMWF-hosted data store, and it entails two prominent drought indices: the Standardized Precipitation Index (SPI) and the Standardized Precipitation Evapotranspiration Index (SPEI). Both indices are calculated over a range of accumulation periods from 1 month to 4 years and are available for the full ERA5 climatology from 1940 to today. It also contains validation data that indicates the quality of these drought indices. The ERA5–Drought dataset serves as a valuable tool for environmental agencies and supports sectors such as water management and agriculture, thus contributing to efforts that monitor water and food security.

## Background & Summary

Drought has long been recognized as one of the costliest natural hazards faced by societies^1^. There exist many different definitions of drought: meteorological drought is defined as a period of deficient precipitation, which in turn can induce agricultural and hydrological droughts that affect e.g., soil moisture, discharge, groundwater and reservoir storage. Hence, droughts can have very significant impacts on ecosystems, economies, and human livelihoods. The impact of a drought strongly depends on the time scale during which deficits accumulate^2^. It is generally hypothesized that the longer a drought persists, the bigger and more far-reaching the impacts are^3^. Prolonged precipitation deficits can cause water scarcity during which human water demands cannot be met, which can lead to food insecurity, cause social unrest and instability, and in extreme cases enforce migration.

The frequency and severity of drought events have intensified over the last century and are further expected to intensify as we progress into an even warmer future^4,5^. Human activities, such as deforestation and unsustainable water use, and their feedback on the climate can further cause an exacerbation of drought events^6–9^. While droughts are occurring globally, highly-vulnerable and low and least development countries in particular experience an increasing drought risk that threats socio-economic well-being and puts livelihoods at risk^10,11^. Many of these countries lack well-developed water monitoring systems that enable integrated water resource management to accommodate the needs of communities and the economy in times of scarcity. A global archive of drought events that extends far back into the history could further help to compare and evaluate drought events, study their impact and elucidate trends. Social, economic, and scientific sectors would benefit from an extensive, global drought archive that is freely accessible and facilitates the identification of drought events, their extents, severity and trends over the last century.

Historical drought conditions can be assessed using weather variables recorded at weather stations or through remotely sensed datasets^12^. However, regions with low monitoring station density may suffer from unreliable estimations of drought due to limited data availability. The same holds for observations from synoptic stations that are interpolated to a global grid, such as the dataset from the Global Precipitation Climatology Centre (GPCC^13^). Remote sensing data provide a global alternative, especially if they can cover a long time record. The National Aeronautics and Space Administration’s Tropical Rainfall Measuring Mission (TRMM; active from 1997 to 2015^14^) and the follow-up Global Precipitation Measurement mission (GPM; active since 2014^15^) have been used in conjunction with ground-based radar and rain gauge networks to establish a global long-term history of precipitation estimates since 1979. Similarly, the Global Precipitation Climatology Project (GPCP^16^) and the Climate Prediction Center’s Morphing Technique (CMORPH) from the National Oceanic and Atmospheric Administration^17^ are just a few examples of operational efforts that provide long-term precipitation records. These records extend only back to 1979 and 1998 respectively, since satellite data build their foundation. Despite the fact that these datasets are fundamental assets for monitoring precipitation, they are not without inaccuracies and challenges connected to inter-calibration across sensors, as well as omission and commission errors^18^. Finally, these datasets provide only a partial picture of the water balance at the Earth’s surface as they only observe rainfall and miss the direct dependencies of drought on temperature through, for example, atmospheric demand, which can further exacerbate drought conditions.

Global reanalyses — retrospective analyses of numerical weather prediction models generated from initial estimates of climate conditions and refined with observations^19,20^ — offer a more consistent spatial and temporal representation of water fluxes in the Earth system for drought assessment. Reanalyses provide a coherent picture of all atmospheric variables which facilitate the definition of multiple drought indices. For example, reanalysis-based drought indices can account for the loss of water from the land surface through evapotranspiration and enable the quantification of atmospheric water demand (often expressed as potential evapotranspiration in which the land surface can supply enough moisture to meet the atmospheric demand). While errors and uncertainties associated with a reanalysis are sometimes not fully assessed, there are several studies that demonstrate how reanalyses can provide reliable information for most parts of the world^21^. For this reason reanalyses are commonly employed as a proxy for observational data^22–24^. Moreover, reanalysis data usually cover multiple decades and the entire globe, making them an invaluable resource for examining long-term statistical patterns and trends beyond observational records. Thus, the limitations imposed by sparse monitoring networks can be overcome by leveraging reanalysis datasets. Using reanalyses allows to not only monitor droughts globally, but also creates an extensive historical database that extends further back into the last century, enabling us to gain insights into global drought occurrence and evolution.

This paper describes the ERA5–Drought dataset — a reanalysis-based dataset of drought indices using the ECMWF Reanalysis version 5 (ERA5). ERA5 provides key variables required to calculate several drought indices^25^ to a resolution of 0.25° globally (around 28 km) from the start of the reanalysis in 1940 to today^26^. The reanalysis consists of 1 deterministic and 10 ensemble members, enabling the quantification of uncertainties arising from slightly different initial conditions in the simulation of the numerical weather prediction model. We believe that this dataset is a valuable resource for applications in many sectors, from the agricultural sector^27^, agencies and scientists in the field of food security and water scarcity^28^, and beyond. As this dataset utilises the same meteorological inputs as other hazard and impact-based datasets for, e.g., fire^24^, heat^29^, and flood^30^, it also facilitates the analysis of compound and cascading events in the Earth system, as well as the impact of drought on global and local resources and populations.

## Methods

This study uses the latest ECMWF reanalysis database ERA5^26,31^ to calculate a set of drought indices. Here, the focus is set on two most commonly used drought indices: the Standardized Precipitation Index (SPI,^2^) and the Standardized Precipitation Evapotranspiration Index (SPEI,^32^), but the dataset may expand in the future to include more indices based on the availability of fields for their calculation in ERA5. The methodology used to calculate the drought indices is described below.

### Drought indices

#### Standardized Precipitation Index (SPI)

The SPI^2^ is a widely used drought index that quantifies precipitation deficits over various time scales. The SPI provides a standardized measure of precipitation anomalies, making it suitable for comparing drought severity across different regions with varying climatic conditions.

**Fig. 1:**
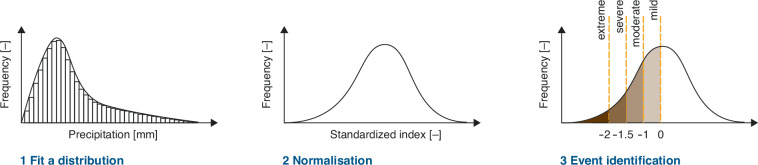
The three steps to calculate a standardised precipitation index (SPI): first, a time series of precipitation is extracted and precipitation is accumulated over, e.g., 1, 3, 6, 12, 24, 36, 48 months using a moving window. Second, a parametric statistical distribution is fitted to the time series for each calendar month (e.g., the Gamma distribution), and the probabilities from this fitted distribution are transformed to the standard normal distribution. Third, the resulting index values indicate the number of standard deviations from the long-term mean and make it comparable in time and space.

The general procedure for calculating the SPI as introduced by^2^ is as follows (see Fig. 1): First, a time series of precipitation for each point (i.e., grid point) is extracted and precipitation is accumulated over the previous *n* months using a moving window. A moving window is applied to obtain a value for each month, always considering the accumulation over the previous *n* months (hereafter termed accumulation period). Then a parametric statistical distribution is fitted to the time series for each calendar month. The Gamma distribution is often used in the calculation^2,33,34^, but requires the removal of months without precipitation (i.e., *P* = 0 *mm*). Second, the probabilities from this fitted distribution are transformed to the standard normal distribution (z-distribution with *μ* = 0 and *σ* = 1). The resulting index values indicate the number of standard deviations from the long-term mean (climatology) and make it comparable in time (e.g., for different calendar months) and space. Finally, individual time steps are then interpreted as anomalies with respect to the standardized distribution. Negative values indicate enhanced precipitation deficits with respect to the climatology and dry anomalies; positive values indicate precipitation surplus with respect to the climatology and wet anomalies. The strength of these wet and dry anomalies depends on the magnitude of the index and can be categorised (see Tables 1+ 2).

**Table 1:** Categorization of the severity of wet and dry events based on the SPI values.

Index value	Categorization	Probability [%]
≥2.00	extremely wet	2.3
1.50 to 1.99	severely wet	4.4
1.00 to 1.49	moderately wet	9.2
0 to 0.99	near-normal/mildly wet	34.1
−0.99 to 0	near-normal/mildly dry	34.1
−1.49 to −1.00	moderately dry	9.2
−1.99 to −1.50	severely dry	4.4
≤−2.00	extremely dry	2.3

**Table 2:** Alternative categorization of the severity of wet and dry events often used for SPEI values.

Index value	Categorization	Probability [%]
≥2.33	extremely wet	1
1.65 to 2.32	severely wet	4
1.28 to 1.64	moderately wet	5
0.84 to 1.27	mildly wet	10
−0.83 to 0.83	near-normal	60
−1.27 to −0.84	mildly dry	10
−1.64 to −1.28	moderately dry	5
−2.32 to −1.65	severely dry	4
≤ − 2.33	extremely dry	1

This procedure can be applied to any time series of precipitation over different timescales, such as sub-annual accumulations of 1-, 3-, and 6 months, or multi-annual accumulations of 12-, 24-, 36-, and 48 months. This procedure is performed for every month of the record, i.e. a fit is performed for every calendar month and every accumulation period. However, in regions with many months or accumulated time periods without precipitation it is difficult to make a robust estimation of the parameters of the gamma distribution. Many previous studies use the historical occurrence of zero precipitation to adjust the SPI values. I.e., the probability of encountering zero (${p}_{0}=\frac{{n}_{P=0}}{n+1}$ within a sample of length *n* and *n*_*P*=0_ entries being 0) is used to calculate the SPI value for these months as follows:

$$p(x)={p}_{0}+(1-{p}_{0})F({x}_{p > 0},\lambda )$$

where *p* is the probability distribution for precipitation *x*, and *F*(*x*, *λ*) is the parametric probability distribution fitted to the time series of non-zero precipitation. However, this can result in a skewed distribution, whose mean is not 0 anymore (see^35^). To adjust for this shift^35^ suggested to adjust the probability of zero precipitation using the centre of mass of the zero distribution instead of the maximum probability:

$$\bar{{p}_{0}}=({n}_{P=0}+1)/(2\ast (n+1)),$$

where $\bar{{p}_{0}}$ is the center-of-mass-corrected mean probability of multiple zeros (see^35^).

A record of at least 30 years is generally recommended to get reliable and statistically significant drought indices. Using the categorisation from^2^ (see Table 1), moderate drought conditions occur if the SPI is between  − 1 and −1.5, severe drought conditions occur if the SPI is between -1.5 and -2.0, and extreme drought conditions occur if the SPI is smaller than -2.0. Severely dry conditions occur by definition 4.4% of the time, and extremely dry conditions occur only 2.3% of the time, if the entire time series is used to fit a distribution. Values much larger or much lower than 3 and -3, respectively, should be interpreted with caution. As these values are on the edge of the distribution and occur very rarely, they come with a large uncertainty (for more details see, e.g.^35^) and should not be interpreted as exceptionally extreme; thus values larger than 3 or lower than -3 should simply be treated as extreme events.

In ERA5–Drought, precipitation from ERA5 is used and accumulated over 1, 3, 6, 12, 24, 36, 48 months to calculate the SPI. The Gamma distribution is then fitted to the precipitation accumulations analogous to^2^,^35^. The period 1991–2020 is used as reference period as recommended by the World Meteorological Organisation (WMO). A distribution is fitted if more than ten entries from that record are not zero; and the quality of that fit is evaluated in a second step (see Section Quality Criteria below). Months and accumulation periods without precipitation are corrected using the adjusted probability of zero precipitation^35^. The full climatology from the ERA5-based precipitation accumulations over 1, 3, 6, 12, 24, 36, 48 months since 1940 are then analysed with respect to that standardized distribution to obtain the SPI values.

#### Standardized Precipitation Evapotranspiration Index (SPEI)

The SPEI integrates both precipitation and potential evapotranspiration (PET) data into drought assessment^32^. The SPEI captures the combined effect of water availability (approximated by precipitation) and atmospheric water demand on drought conditions. The integration of land surface processes impacts the accuracy of drought assessment, especially in arid and semi-arid regions where evaporation plays a significant role in water balance dynamics.

The computation of SPEI involves the estimation of PET which represents the amount of water that would evaporate and transpire from a hypothetical reference surface under prevailing meteorological conditions. Various empirical and physically based methods are available for estimating PET, such as Hargreaves^36^, Thornthwaite^37^, Priestley-Taylor^38^, and Penman-Monteith^39–42^, among others and combinations of those. Especially the Hargreaves and the Thornthwaite parameterisation are often used as a first proxy of PET due their limited input data requirements. Studies indicate that the SPEI is very sensitive to the parameterisation used^43,44^ but most studies concur that the physically-based Penman-Monteith parameterisation should be used, if possible. The Penman-Monteith equation estimates potential evapotranspiration as a function of net radiation at the surface of a hypothetical crop, water vapor pressure deficit (*e*_*s*_ − *e*_*a*_), soil heat flux, and the slope of the temperature-saturation vapor pressure relationship as

1 $$PET=\frac{0.408\cdot \Delta \cdot ({R}_{n}-G)+\gamma \cdot \frac{900}{T+273}\cdot {u}_{2}\cdot ({e}_{s}-{e}_{a})}{\Delta +\gamma \cdot (1+0.34\cdot {u}_{2})}$$

where:

$$\begin{array}{l}PET={\rm{Potential\; evapotranspiration}}\,\,({\rm{mm}}/{\rm{day}})\\ \,\Delta ={\rm{Slope\; of\; the\; saturation\; vapor\; pressure\; curve}}\,\,(\,{\rm{kPa}}\,{/}^{\circ }{\rm{C}})\\ \,{R}_{n}=\,{\rm{Net\; radiation\; at\; the\; crop\; surface}}\,\,({\rm{MJ}}/{{\rm{m}}}^{2}/{\rm{day}})\\ \,G=\,{\rm{Soil\; heat\; flux\; density}}\,\,({\rm{MJ}}/{{\rm{m}}}^{2}/{\rm{day}})\\ \,\gamma =\,{\rm{Psychrometric\; constant}}\,\,({\rm{kPa}}{/}^{\circ }{\rm{C}})\\ \,T=\,{\rm{Mean\; daily\; air\; temperature\; at\; 2\; meters\; height}}\,{(}^{\circ }{\rm{C}})\\ \,{u}_{2}=\,{\rm{Wind\; speed\; at\; 2\; meters\; height}}\,({\rm{m}}/{\rm{s}})\\ \,{e}_{s}=\,{\rm{Saturation\; vapor\; pressure}}\,\,({\rm{kPa}})\\ \,{e}_{a}=\,{\rm{Actual\; vapor\; pressure}}\,\,({\rm{kPa}}),\end{array}$$

and hence requires a lot of input data that is often not available from observations.

The SPEI is computed by subtracting potential evapotranspiration from precipitation (i.e., *P* − *PET*), and indicates the theoretical surplus or deficit of water at the land surface, balancing water supply and atmospheric water demand. Analogous to the SPI, a distribution is fitted to the climatology of *P* − *PET* values, and standardized to obtain the SPEI values that enable the assessment of drought severity relative to the long-term climatic variability. Vicente-Serrano *et al*.^32^ suggested to use the log-logistic distribution, which has also performed well in many other studies (e.g.^45,46^). As *P* − *PET* is barely identical to 0, no modifications analogous to the SPI are required.

**Fig. 2:**
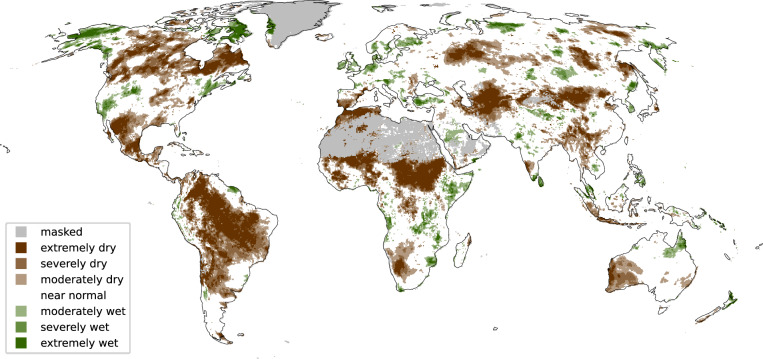
Example from the data record. Global snapshot of the SPEI-12 at the end of December 2023. The global map shows regions that have experienced dry (brown) and wet (green) conditions in 2023 according to the SPEI, i.e. the difference between precipitation and atmospheric water demand (expressed as potential evapotranspiration). The intensity of the dry and wet conditions is categorized as moderate, severe, and extreme and is shown as different shadings of brown and green, respectively. The intensity is evaluated with respect to the reference period 1991–2020. This map highlights the vast long-term drought that extends over large parts of South America that has been affecting people living in Brazil and neighbouring countries in the last years. However, also other countries in Africa and Central America, Canada, and Central Asia have experienced severe drought conditions. Regions without vegetation (deserts and polar regions) or insufficient quality are masked grey.

In ERA5–Drought, the Penman-Monteith parameterisation is used to estimate potential evapotranspiration to compute the SPEI. The log-logistic distribution is fitted to *P* − *PET* accumulations from 1991–2020, again following the WMO recommended reference period, before standardizing the values. The full climatology from 1940–today is then analysed with respect to that standardised distribution to obtain monthly SPEI values. Analogous to the SPI, accumulation periods of 1, 3, 6, 12, 24, 36, and 48 months are considered. Figure 2 illustrates a snapshot of the resulting SPEI-12 at the end of December 2023.

### Quality criteria

In addition to the drought indices we perform a quality control of the derived dataset. The quality is assessed by testing if the distribution of the estimated drought indices over the reference period follows a normal distribution with mean 0 and standard deviation 1. The test is performed using the Shapiro-Wilks test for normality^47^ with *α* = 0.05. This quality test is performed for both drought indices and every calendar month using the derived indices during the reference period (1991–2020). The resulting p-value of the Shapiro-Wilks test is checked and the corresponding quality parameter is set to 0 if the p-value is below *α* = 0.05, and set to 1 if it exceeds *α*.

For the SPI, the probability of zero precipitation (’pzero’) is also provided. While the Gamma distribution is fitted to the historical record if at least ten values are not zero, this second quality criterion enables the definition of reasonable thresholds, and the use of drought indices in region with less than 30 months of precipitation larger than 0.

## Data Record

The ERA5-Drought dataset is available through the ECMWF-hosted Cross Data Store (XDS) via 10.24381/9bea5e16^48^.

ERA5–Drought version 1.0 consists of the SPI and the SPEI calculated over a range of accumulation periods (1, 3, 6, 12, 24, 36, 48 months), where the accumulation period is indicated in the name of the drought index. For example, an SPI calculated with an accumulation period of 1 month is denoted as SPI-1, and an SPI calculated with an accumulation period of 12 months is denoted as SPI-12, and so on. The valid date of both drought indices is set to the end of each accumulation period, e.g., the SPI-12 in January 2024 uses the previous 12 months. All drought indices are available for each calendar month for which ERA5 data is available — i.e., from 1940 to today. However, drought indices with accumulation periods larger than 1 month start with a corresponding delay. E.g., the SPI-12 only starts in January 1941, as the previous 12 months are required for the accumulation.

We used two products from the ERA5 dataset to calculate drought indices: the deterministic model output (simply called “reanalysis" in most applications), and the probabilistic model output that consists of 10 ensemble members. Both SPI and SPEI are calculated for each product, rendering a deterministic and a probabilistic version of each drought index. The latter ensemble allows to assess uncertainties associated with ERA5 data.

At the time of writing, both ERA5 and ERA5–Drought data are available from January 1940 to December 2024 (referred to as the “Consolidated dataset”). As the process of consolidating and releasing the official dataset takes about two months, there also exists an “Intermediate dataset” of ERA5, also referred to as ERA5T. This experimental dataset is published behind real-time and could be affected by changes before the official release date. An intermediate release is also issued for the ERA5–Drought dataset, enabling a near real-time monitoring of droughts with just one month of delay. However, the ERA5–Drought dataset is updated with each update of the consolidated ERA5 dataset as well.

### Data format

ERA5–Drought is made available on a regular unprojected grid with spherical coordinates expressed in decimal degrees (EPSG:4326). Latitudes span the range from -90 to +90 degrees and are referenced to the equator. Longitudes are in the range from -180 to 180 degrees, referenced to the Greenwich Prime Meridian, consistent with ERA5^31^ and derived products that use ERA5 meteorological fields for other application such as fire prediction^24^ or heat stress^29^. The spatial resolution is 0.25° × 0.25° (about 28 km grid cell size).

Data are provided in netCDF format following the CF-standard v1.6 convention and for each drought index (SPI, SPEI), accumulation window (1, 3, 6, 12, 24, 36, 48), distribution (gamma for SPI, log-logistic for SPEI), dataset (ERA5 for the consolidated dataset or ERA5T for the unconsolidated dataset), stream (moda for the deterministic data stream, edmo for the probabilistic data stream, both following the ERA5 stream naming convention), and year and month separately. The files are named as follows:


(index)(window)_(distribution)_global_(dataset)_(stream)_ref1991to2020_(yyyy)(mm).nc.


The file naming for the quality criteria (pzero, significance) is analogous and follows the structure:


(index)(window)_(index) (quality_criterion)_(distribution)_global_(dataset)_(stream)_ref1991to2020_(mm).nc.


Quality criteria are calculated for the reference period (i.e., 1991–2020) but only one time step and file for each month is provided (thus omitting the year in the filename).

## Technical Validation

### Data quality criteria

**Fig. 3:**
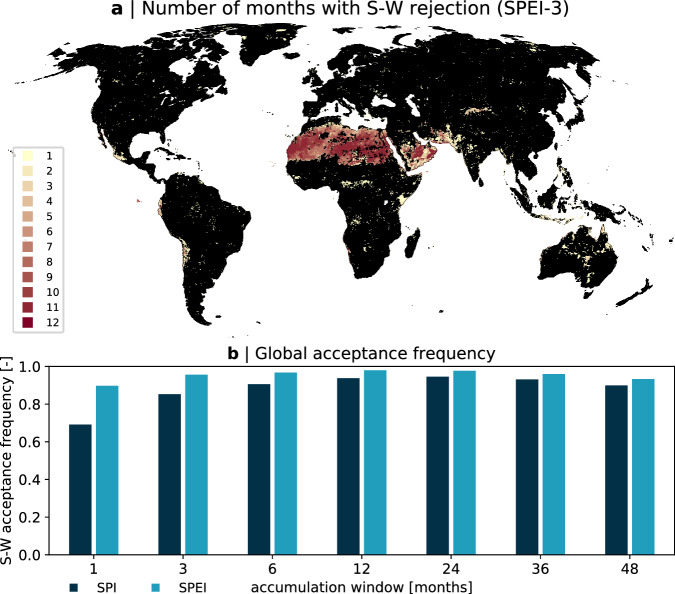
Quality of derived drought indices. (**a**) Global distribution of number of months during which the Shapiro-Wilks test for normality indicates a rejection of the null hypothesis for SPEI-3 (*α* = 0.05). Black colored regions indicate full reliability. (**b**) Global acceptance frequency calculated over all 12 months and all land pixels (including desert and polar regions) for the SPI (dark blue) and the SPEI (light blue) for all accumulation windows considered.

The quality of the standardized drought indices is expressed as the fraction or percentage of all land pixels and all months during which the Shapiro-Wilks test indicates normality at the 5% significance level. Figure 3 shows a map of hot spots where the quality criteria are not met and a bar chart that evaluates the global quality for both indices and various accumulation windows. The corresponding global acceptance is above 90% for all SPEI indices, and for all SPI indices with accumulation windows larger than three months (see Fig. 3b). The SPI is generally less reliable than the SPEI (compare dark and light blue in Fig. 3b). The lowest reliability is found for small accumulation windows: only 69% and 90% of all land pixels and all months pass the test for normality at 5% for the SPI-1 and the SPEI-1, respectively.

The largest rejection rates are found over the Sahara and the Arabian peninsula. For the SPI with small accumulation periods of one and three months, also south Asia and central and north Australia indicate a lower reliability. If desert and polar regions are excluded, the acceptance frequency for the SPI-1 and the SPEI-1 increases to 72% and 94% (not shown), respectively.

This result is expected as especially the SPI is subject to the occurrence of months without precipitation. While this can be accounted for (see description above), it can result in a skewed distribution that does not follow a gamma distribution anymore and hence the fit and the standardization fail. However, we find that the mean acceptance rate for the SPI-1 increases to 76% if regions with more than 3 months with zero precipitation in the reference period are excluded, i.e., only regions where pzero  < 0.1 are retained. While many studies require at least 30 values other than 0, we intentionally tested these thresholds here and provide also drought indices for regions and months with less data availability for exploration. Globally, we find only minor improvements of the acceptance rate as regions with many months of zero precipitation are excluded — however, we suggest to mask the SPI in regions where pzero  > 0.1 (i.e., more than 3 out of 30 values are zero) to increase the reliability of the dataset. In addition, we generally also recommended to mask desert and polar regions for both SPI and SPEI.

It is noted that also other distributions were tested during the calculation of the SPI and the SPEI, but the Gamma and the generalized logistic distribution perform best globally (not shown).

### Comparison with other datasets

As drought indices are not observable quantities, the ERA5–Drought indices are validated against other global and operational products of the same indices.

The Copernicus Global Drought Observatory (GDO) provides a global SPI dataset with accumulation windows of 1, 3, 6, 9, 12, 24, and 48 months that is available from 1981 to today. This SPI uses precipitation from GPCC (http://gpcc.dwd.de/) that is a monthly, gridded precipitation dataset based on measurements from surface synoptic stations that are interpolated to a 1° grid. The “first guess” product from GPCC is used for the most recent two months and then updated using the “Monitoring" product. At the time of writing, the reference period for the SPI dataset from GDO is 1981–2020. Through the web portal from GDO, this dataset can be downloaded as GeoTiff or netCDF files (see https://drought.emergency.copernicus.eu/tumbo/gdo/download).

The Spanish National Research Council (Consejo Superior de Investigaciones Científicas, CSIC) services a global SPEI product via their operational drought monitoring system and a corresponding drought database (see https://spei.csic.es^32,44,49^). The CSIC drought monitor provides monthly updates of the SPEI with accumulation periods between 1 and 48 months (i.e., all 48 accumulation periods, from 1, 2, 3, … to 48 months) at 1° resolution. For the calculation of this near real-time SPEI, a reference (calibration) period from 1955–2010 is used (and extended to 1950 for the 4-year accumulation windows). Near real-time updates are provided using the ’First guess’ product from GPCC and mean temperatures from NOAA’s Global Historical Climatology Network version 2 and the Climate Anomaly Monitoring System (GHCN + CAMS) gridded dataset to estimate potential evapotranspiration using the Thornthwaite parameterisation. This 1° estimate of the SPEI near-real time is available from 1955 to today. In addition to this drought monitoring dataset, CSIC provides a more robust drought database that is based on the Penman-Monteith parameterisation of potential evapotranspiration, and uses a higher spatial resolution. The most recent version of the Drought database, SPEIbase v2.9 (https://spei.csic.es/spei_database_2_9), uses gridded observations from the Climatic Research Unit (CRU) of the University of East Anglia TS 4.07 dataset as input and is available at 0.5° spatial resolution for all calendar months from January 1901 to December 2022, and for all accumulation periods between 1 and 48 months.

As comparable drought indices are only available at coarser resolutions than ERA5–Drought (i.e., at 1° and 0.5°, while ERA5–Drought is available at 0.25°), only a qualitative validation is performed. This validation is designed to ensure that the ERA5–Drought data record is free from clerical errors. However, as both GDO and CSIC also use other input data, make other assumption and use other reference periods, differences may arise from the impact of these factors on the estimation of the drought indices. An overview of the major differences are provided in Table 3.

**Table 3:** Overview of other global SPI and SPEI datasets and their differences.

	GDO	CSIC	ECMWF
**Indices**	SPI	SPEI	SPI, SPEI
**Accumulation window**	1, 3, 6, 9, 12, 24, 48 months	1, 2, 3, …, 48 months (all months)	1, 3, 6, 12, 24, 36, 48 months
**Lineage**	P from GPCC	P from GPCC and T from NOAA (monitor), P and T from CRU (database)	P and PET from ERA5
**Reference Period**	1981-2010	1955-2010 (extended to 1950 for 4-year accumulation windows)	1991-2020
**Temporal availability**	1981-today (2-3 months delay)	1955-today (monitor, 1 month delay), 1901 to 2022 (database, v2.9)	1940-today (1 month delay for intermediate data, 2-3 months delay for consolidated data)
**Spatial resolution**	1.0°	1.0° (monitor), 0.5° (database)	0.25°
**Uncertainty estimates**	N/A	N/A	based on ensemble of drought indices
**Quality assurance**	only input data	only input data	input data and indices (Shapiro-Wilks test for normality)
**Accessibility and license**	free (CC-BY 4.0), manual downloads via website	free (ODbL 1.0), manual downloads via website	free (CC-BY 4.0), downloads via XDS, either through the website or using an API in python

Note that GDO provides other drought indices as well, but that the information in this table is focused on the SPI as available on their website.

The SPI and the SPEI from ERA5–Drought are compared with the SPI and SPEI from GDO and CSIS, respectively. The focus is set on two major drought events in different climates: the most recent drought over Brazil and across South America, and Australia’s Tinderbox drought between 2017–2019^50^.

**Fig. 4:**
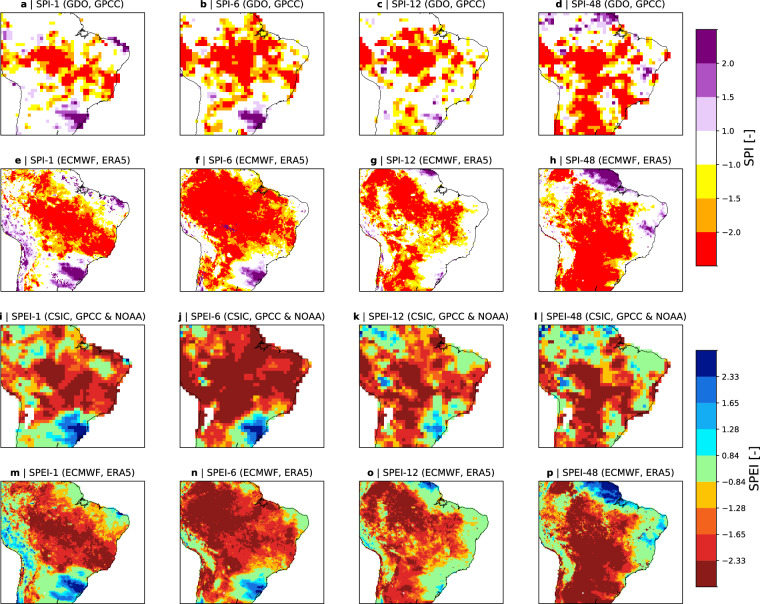
Comparison of the ERA5–Drought dataset to drought indices from GDO and CSIC for Brazil at the end of December 2023. Maps show the SPI-1, -6, -12, and -24 from GDO (**a,b,c,d**) and ERA5–Drought (**e,f,g,h**), and the SPEI-1, -6, -12, -24 from CSIC (**i,j,k,l**) and ERA5–Drought (**m,n,o,p**). Note that the color schemes and thresholds are adjusted to the GDO and CSIC schemes for SPI and SPEI, respectively.The SPEI from CSIC stems from their drought monitor that uses the Thornthwaite equation to estimate potential evapotranspiration and provides the indices at 1°resolution. Non-vegetated areas and non-reliable drought estimates from ERA5–Drought are masked grey.

Figure 4 shows the SPI-6, -12, -24 from GDO and from ERA5–Drought over Brazil at and the SPEI-6, -12 and -24 from CSIC’s drought monitor and ERA5–Drought at the end of November 2023. The CSIC drought monitor data at 1° is chosen here, as their drought data base does not contain any data beyond 2022 at the time of writing. For better comparison with online data, SPI and SPEI maps are plotted using the color schemes and thresholds from GDO and CSIC, respectively.

**Fig. 5:**
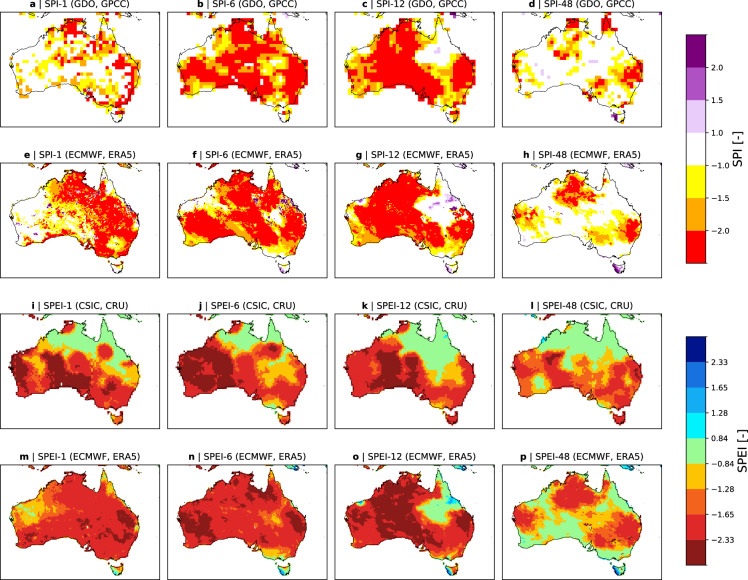
Same as Fig. 4, but for Australia. The SPEI from CSIC stems from their drought data base that uses the Penman-Monteith equation to estimate potential evapotranspiration and provides the index at 0.5°resolution.

ERA5–Drought shows good agreements with the SPI from GDO and the SPEI from CSIC for both drought events. For the recent drought over Brazil, ERA5–Drought indicates slightly larger and more widespread precipitation deficits for all accumulation periods (compare Fig. 4a–d with Fig. 4e–h). The corresponding SPEI from ERA5–Drought also shows an increased area in drought compared to the SPEI from CSIC (compare Fig. 4i–l with Fig. 4m–p), though the differences are less pronounced. The same holds for the Tinderbox drought in Australia, for which ERA5–Drought also indicates a slightly increased area in drought, especially for the SPEI with 1- and 3 month accumulation periods (compare Fig. 5i–j with Fig. 5m,n). However, while differences are expected (see reasons explained above), the general drought patterns and intensities for both drought events are remarkably similar and highlight the similarity of ERA5–Drought to existing datasets.

### Known limitations from ERA5

A key difference in the above depicted drought indices stems from the underlying datasets, which are gridded observations or reanalyses and come with their own limitations. In ERA5, one notable limitation pertains to the pre-satellite era, particularly before 1980. During this period, the scarcity of observational data leads to increased uncertainty in the reanalysis outputs. A study by^51^ highlights that the number of assimilated observations in ERA5 increases from 53,000 per day in early 1950 to 570,000 per day by the end of 1978, indicating a significant improvement in data availability over time. Consequently, the quality of the reanalysis improves throughout this period, generally joining seamlessly with the segment covering 1979 to the present. Additionally, data prior to 1960 may be more strongly influenced by the initial conditions used in the reanalysis model, as the limited observational constraints allow model biases and spin-up effects to have a greater impact. Therefore, users should interpret early reanalysis data with caution, particularly when analyzing long-term trends or extreme events.

Precipitation in ERA5 has been evaluated against in-situ observations and other gridded datasets. Studies have found that ERA5 generally provides a realistic representation of precipitation patterns, but discrepancies can occur, especially in regions with complex topography or sparse observational networks. For instance^21^ conducted a validation study comparing ERA5 precipitation data with in-situ observations across Europe and found that while ERA5 captures the overall precipitation distribution well, extreme precipitation events tend to be underestimated.

Potential evaporation is derived from various parameters in ERA5. In addition to the uncertainties stemming from the parameterization to calculate potential evaporation, the accuracy of the input variables plays a role. In terms of temperature, ERA5 has been shown to perform well in capturing surface and atmospheric temperature variability. However^52^ found that the ERA5 temperatures performed better in temperate regions compared to tropical regions when validated against land-based observations from the Global Historical Climatology Network. Additionally, during heatwave events, ERA5 may underestimate maximum temperatures due to its spatial resolution and the smoothing inherent in reanalysis processes.

In summary, while ERA5 represents a significant advancement in reanalysis data, especially with its high spatial and temporal resolution, users should be mindful of its limitations. The accuracy of variables such as precipitation and potential evaporation can vary depending on the region, time period, and the availability of observational data. While some uncertainties of the derived drought indices can be addressed through the provided 10-member ensemble (’edmo’ stream), additional uncertainties due to biases in the input variables of SPI and SPEI may be present.

## Usage Notes

This section provides details on how to access the ERA5–Drought dataset and illustrates usage examples, as the dataset offers insights for a range of applications. For example, users can analyse time series of drought occurrence and severity for specific locations or undertake more intricate analyses, such as exploring the impact of drought on regions and local communities. In the examples that follow we demonstrate how such tasks can be accomplished with ease using the ERA5–Drought dataset, focusing on two additional drought-affected regions: Catalonia (Spain) and Kenya.

### Data access

As described in the Data Record section, the dataset is accessible via the Cross Data Store hosted by ECMWF. The ERA–Drought dataset, like any other dataset included in one of the ECMWF-hosted data stores, is subject to the CC-BY 4.0 license. This license follows a full free and open data policy, allowing anyone, anywhere in the world, to access, share, use and adapt the data. Users are only asked to give appropriate credit and indicate if changes were made.

The landing page provides an overview of the dataset, its availability and metadata. Under the tab ’Download data’, users can explore the dataset through a dynamic web interface that automatically indicates availability of the selected index for a specific time period and vice versa.

To download data, users need to register under the CC-BY 4.0 license. After login, data can be downloaded via the web interface directly, through the submission of a request, or using an Application Program Interface (API). Codes for the selection of the data via the API or python scripts are provided upon selection and facilitate access to a broad range of users.

### Illustration of use: Time series of area in drought and comparison to previous drought events

**Fig. 6:**
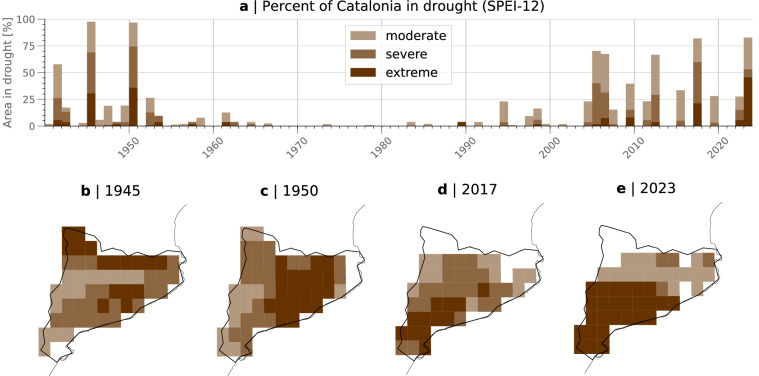
Usage example: Climatology of annual drought events over Catalonia, Spain. (**a**) Time series of the area of Catalonia in moderate/severe/extreme drought (in %, indicated in light/medium/dark brown colors) based on the SPEI-12 for each calendar year between 1940–2023 using the ERA5–Drought dataset. (**b**–**e**) Maps of four of the most extensive annual drought events over Catalonia, i.e., 1945, 1950, 2017, 2023. Near-normal values are masked white (analogous to Fig. 2).

Figure 6 shows a time series and maps of annual drought events over Catalonia from 1940–2023. The time series shows the area experiencing moderate, severe, and extreme drought using the SPEI for each calendar year available from the record; colors indicate the intensity of the drought. The intensity of drought events can be compared using the area in drought (see Fig. 6a) and the spatial distribution of drought severity (see Figs. 6b-f), and exceptionally dry years can be identified. Figure 6 corroborates the severity of droughts in 2004–2007, 2017, and 2023, which have been studied and discussed intensively (e.g.^53,54^).

In addition, the ERA5–Drought data also considers many years between 1940–1960 as dry — while there have been studies that corroborate this finding (e.g.^55–57^), the dry anomalies prior to 1980 may be subject to biases in the back-extension of ERA5 (see limitations described above): the general variability of monthly precipitation from ERA5 shows good correspondence with observations from 1950 onward for all continents^51^. However, in addition to the availability of more conventional observations from 1978 onward, the accuracy of the reanalysis dataset is increasing as satellite data becomes available and helps to monitor the climate globally. Previous studies have shown that precipitation in ERA5 shows the smallest errors in the extra-tropics^21^, but that any reanalysis is subject to biases throughout the production period^58^. The ERA5–Drought dataset relies on the data from ERA5 and is thus subject to the accuracy of this dataset. Drought events identified at the beginning of the data record should hence be considered subject to biases, although a debate about these rather dry years is ongoing.

### Illustration of use: Association with impacts on society

**Fig. 7:**
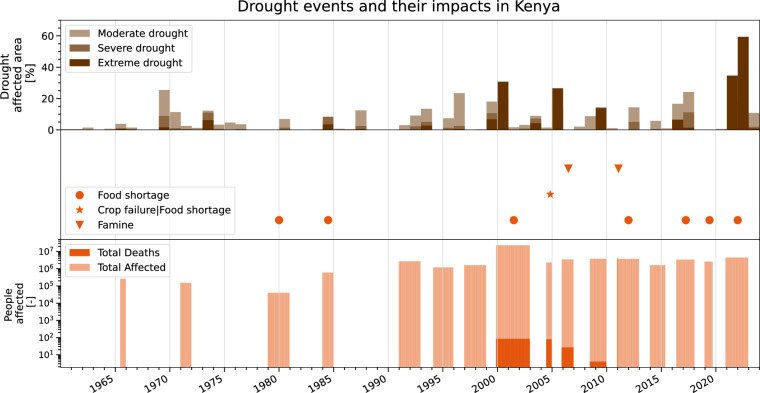
Usage example: Impacts associated with droughts in Kenya. Time series of the area of Kenya in drought (brown bars, same as Fig. 6 but using the SPI-12 for each calendar year) based on ERA5–Drought, and related impacts (orange symbols and orange bars) based on EM-DAT registered drought events. Symbols indicate associated disasters at the country level, i.e., food shortage, crop failure & food shortage, and famine. EM-DAT events of unknown start date and end date are plotted over the full event years; symbols in the center of the event. Data is restricted to 1960–2023 due to the availability of EM-DAT data.

Prolonged droughts can have far-reaching impacts that affect society (e.g.^59^), and especially highly-vulnerable societies are predicted to experience more frequent and more intense water shortages induced by drought^60^. These water shortages affect the availability of drinking water and can further cause crop failure and food insecurity and, in the worst case induce famine and cause deaths^61^. The ERA5–Drought data can be used to associate registered water- and food insecurity events with the occurrence and intensity of meteorological droughts and thus enables applications beyond environmental science. Here, we demonstrate the use of ERA5-Drought in conjunction with impact data from the Emergency Events Database (EM-DAT^62^), which records the occurrence of natural disaster worldwide and compiles their socio-economic impacts from reports and articles published by press agencies, UN-, non-governmental, and other research organizations, among others. Drought events in EM-DAT are registered if 10 or more fatalities were reported, 100 or more people were affected, a state of emergency was declared, or a call for international assistance was issued. While the EM-DAT database is constantly growing, it is not considered to be exhaustive — yet, it enables an estimation of the impact of droughts on society. An analysis of Kenya’s impacts illustrates the co-occurrence of meteorological drought (expressed as the SPI-12) with food insecurity as reported in EM-DAT. Figure 7 shows the area of Kenya affected by droughts as brown bars, the registered impacts as orange symbols (i.e., famine, crop failure & food shortage, and food shortage as orange triangles, stars, and circles, respectively) and the number of people affected as orange bars. This analysis shows that all reported drought impacts occurred during or shortly after meteorological droughts. In particular, ERA5-Drought identifies the years 2021–2022/2023 as extremely dry, which left approx. 50.1 million people food insecure at the end of 2023^63^, thus further supporting the credibility of the ERA5-Drought dataset.

These usage examples demonstrate that ERA5-Drought facilitates a multitude of applications, ranging from the identification, over the evaluation to a contextualization of meteorological drought events under climate change. In conjunction with other socio-economic datasets, ERA5-Drought can be used to, e.g., assess the impact of droughts and the resilience and vulnerability of societies to droughts worldwide.

## Data Availability

The codes to reproduce the figures in this manuscript are available as jupyter notebooks via https://github.com/jkeune/ERA5-Drought_data. In addition, as part of ECMWF’s Code4Earth challenge (see https://codeforearth.ecmwf.int for details), an interactive jupyter book with detailed information on the data and drought storylines with visualisations that explore and explain this drought dataset is being developed: https://ecmwfcode4earth.github.io/tales-of-drought/. At the date of resubmission of this manuscript, the jupyter book is still in development.
